# Phantom-based correction for standardization of myocardial native T1 and extracellular volume fraction in healthy subjects at 3-Tesla cardiac magnetic resonance imaging

**DOI:** 10.1007/s00330-022-08936-8

**Published:** 2022-06-30

**Authors:** Young Joo Suh, Pan Ki Kim, Jinho Park, Eun-Ah Park, Jung Im Jung, Byoung Wook Choi

**Affiliations:** 1 Department of Radiology, Center for Clinical Imaging Data Science, Research Institute of Radiological Sciences, Severance Hospital, Yonsei University College of Medicine, 50–1 Yonsei-ro, Seodaemun-gu, Seoul, Korea; 2 Department of Radiology, Research Institute of Radiological Sciences, Yonsei University College of Medicine, 50–1 Yonseiro, Seodaemun-gu, Seoul, Korea; 3 Phantomics, Inc., Seoul, Korea; 4 Department of Radiology, Seoul National University Hospital, Seoul National University College of Medicine, Institute of Radiation Medicine, Seoul National University Medical Research Center, Seoul, Korea; 5 Department of Radiology, Seoul St. Mary’s Hospital, College of Medicine, The Catholic University of Korea, Seoul, Korea

**Keywords:** Heart, Magnetic resonance imaging, Standardization, Phantoms, imaging

## Abstract

**Objectives:**

To investigate the effect of the phantom-based correction method for standardizing myocardial native T1 and extracellular volume fraction (ECV) in healthy subjects.

**Methods:**

Seventy-one healthy asymptomatic adult (≥ 20 years) volunteers of five different age groups (34 men and 37 women, 45.5 ± 15.5 years) were prospectively enrolled in three academic hospitals. Cardiac MRI including Modified Look - Locker Inversion recovery T1 mapping sequence was performed using a 3-Tesla system with a different type of scanner for each hospital. Native T1 and ECV were measured in the short-axis T1 map and analyzed for mean values of the 16 entire segments. The myocardial T1 value of each subject was corrected based on the site-specific equation derived from the T1 Mapping and ECV Standardization phantom. The global native T1 and ECV were compared between institutions before and after phantom-based correction, and the variation in native T1 and ECV among institutions was assessed using a coefficient of variation (CoV).

**Results:**

The global native T1 value significantly differed between the institutions (1198.7 ± 32.1 ms, institution A; 1217.7 ± 39.9 ms, institution B; 1232.7 ± 31.1 ms, institution C; *p* = 0.002), but the mean ECV did not (26.6–27.5%, *p* = 0.355). After phantom-based correction, the global native T1 and ECV were 1289.7 ± 32.4 ms and 25.0 ± 2.7%, respectively, and CoV for native T1 between the three institutions decreased from 3.0 to 2.5%. The corrected native T1 value did not significantly differ between institutions (1284.5 ± 31.5 ms, institution A; 1296.5 ± 39.1 ms, institution B; 1291.3 ± 29.3 ms, institution C; *p* = 0.440), and neither did the ECV (24.4–25.9%, *p* = 0.078).

**Conclusions:**

The phantom-based correction method can provide standardized reference T1 values in healthy subjects.

**Key Points:**

• *After phantom-based correction, the global native T1 of 16 entire myocardial segments on 3-T cardiac MRI is 1289.4 ± 32.4 ms, and the extracellular volume fraction was 25.0 ± 2.7% for healthy subjects.*

• *After phantom - based correction was applied, the differences in the global native T1 among institutions* became insignificant, and the CoV also decreased from 3.0 to 2.5%.

**Supplementary Information:**

The online version contains supplementary material available at 10.1007/s00330-022-08936-8.

## Introduction

Myocardial tissue characterization by T1 mapping and estimation of native T1 and extracellular volume (ECV) by cardiac magnetic resonance imaging (CMR) are important for diagnosing and predicting the prognosis of various cardiovascular diseases [1–3]. Assessment of the presence and extent of myocardial abnormalities such as interstitial fibrosis using CMR may provide a surrogate endpoint in clinical trials [4]. However, the application of T1 mapping in multi-institutional, large-scale clinical trials is currently limited because the accuracy and precision of T1 mapping values, especially those of the native T1, vary depending on multiple factors [5–8]. For example, imaging sequence, field strength, temperature, manufacturer-specific hardware design of the CMR system, and even installation sites of CMR can affect estimated values from T1 mapping and normal reference ranges.

Therefore, the consensus statement from the society of cardiovascular magnetic resonance recommends that a local reference range from healthy controls or patients without other signs or history of myocardial disease should be primarily used for native T1 mapping [1]. However, even site-specific T1 measurement values cannot allow direct comparison and integration of results between different sites or CMR systems. To date, several methods have been adopted to standardize the analysis and reporting of T1 and ECV, such as the use of a phantom-based quality assurance system [9], z-score normalization [10], and clustered structuring [11]. However, none of these methods has been accepted as a sole strategy for the standardization of T1 mapping in a clinical setting. Recently, phantom-based quality assurance has been suggested for standardized myocardial T1 measurement [12, 13]. Consistent T1/T2 relaxation time can be obtained for each tube of T1 Mapping and ECV Standardization (T1MES) phantom if chemical stability and temperature dependence are guaranteed. Therefore, we hypothesized that correction of T1 and ECV from T1 mapping sequences of healthy human subjects based on the T1 from a standardized phantom might reduce variation of measurements.

Therefore, we aimed to investigate the effect of the phantom-based correction method for standardizing myocardial T1 measurement in healthy subjects from multiple institutions and provide standardized reference values of myocardial native T1 and ECV.

## Materials and methods

### Study population

**Fig. 1:**
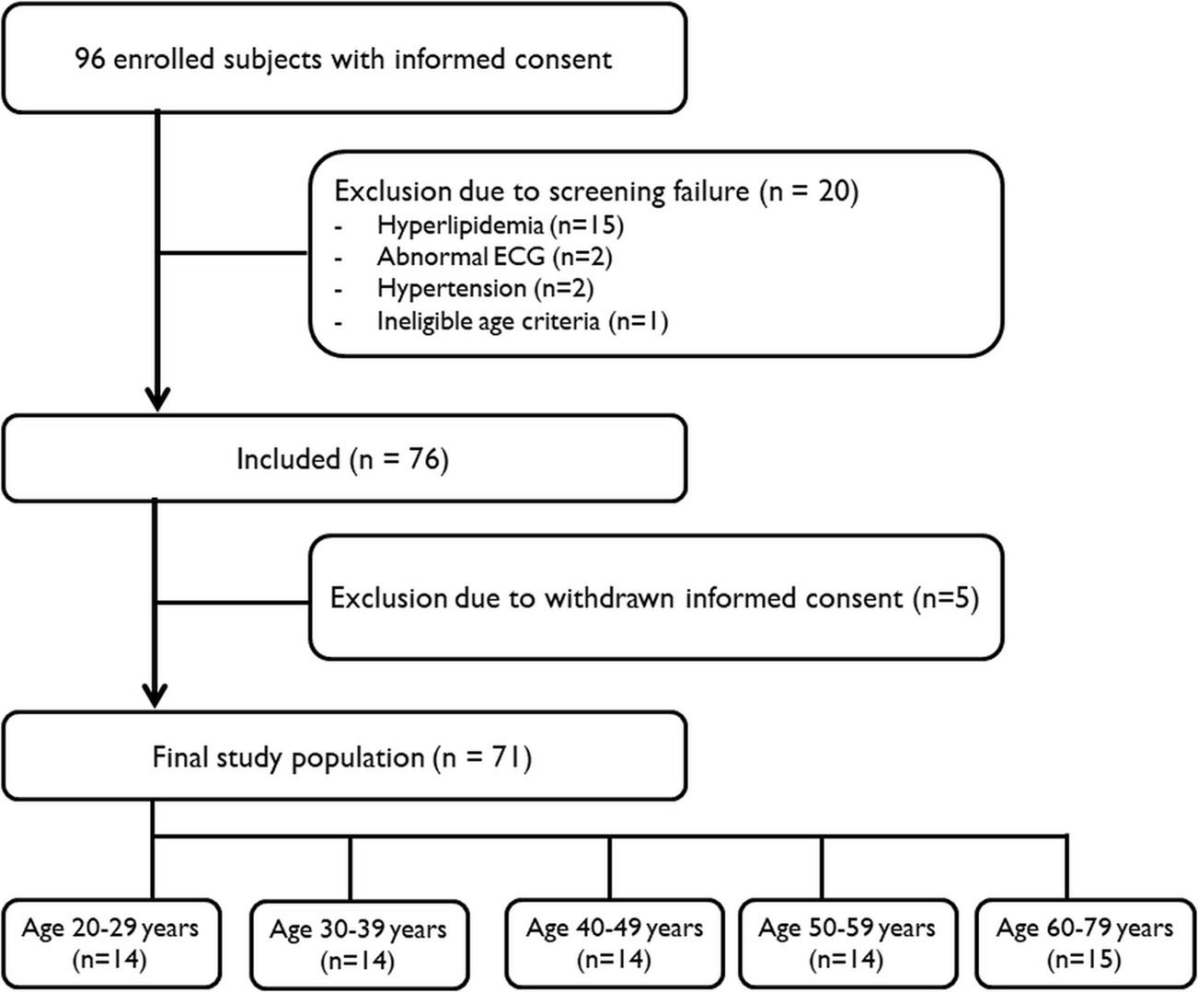
Flow diagram of subject enrollment. ECG, electrocardiogram

This prospective study was approved by the institutional review boards of the participating hospitals. Subjects were enrolled after obtaining written informed consent to participate in this study. From December 2019 to April 2021, healthy asymptomatic adult ( ≥ 20 years) volunteers of five different age groups (20–29 years, 30–39 years, 40–49 years, 50–59 years, and 60–79 years) were prospectively enrolled in three academic hospitals (Fig. 1). The target number of subjects was 14 (7 males and 7 females) per age group, which was determined by referencing the number of healthy subjects in similar researches (9–11 subjects per age group) [14, 15]. Prior to inclusion and CMR, all subjects underwent a clinical examination for symptoms of cardiovascular disease and assessment of medical history and cardiovascular risk factors, such as smoking, hypertension (systolic and diastolic blood pressure > 90mmHg/90 mmHg with home - based remedies or drug treatment), hyperlipidemia, atrial fibrillation, diabetes mellitus, obesity (BMI > 30 kg/m^2^), and family history of cardiovascular disease (acute coronary syndrome or coronary revascularization in first - degree relatives < 65 years old). For screening, all subjects underwent ECG and blood sampling with measurement of estimated glomerular filtration rate (eGFR, Modification of Diet in Renal Disease formula), hematocrit, cholesterol, and N-terminal pro - brain natriuretic peptide prior to inclusion. Exclusion criteria were (1) any evidence of heart disease as indicated by clinical history or physical examination, (2) presence of abnormal ECG or hyperlipidemia (total cholesterol > 240 mg/dL) on screening, (3) pregnancy, (4) contraindications to CMR or expectation of having degraded CMR image quality (ferrometallic cerebral aneurysm clips, pacemaker or implantable defibrillator, or severe claustrophobia), or contraindications to injection of gadolinium-based contrast agent (renal insufficiency with eGFR < 45 mL/min/1.73m^2^), and (5) diabetes. After 96 healthy Korean subjects were screened, 20 subjects were excluded due to screening failure (15 with hyperlipidemia, 2 with abnormal ECG, 2 with hypertension, and 1 who did not meet the age criteria), and 5 subjects withdrew their informed consents after study participation. The final population consisted of 71 participants (34 males, mean age 45.5 ± 15.5 years; 29 subjects from institution A, 16 subjects from institution B, and 26 subjects from institution C), with 14 subjects (7 males and 7 females) per age group between 20–29 and 50–59 years and 15 subjects (6 males and 9 females) in the age group of 60–79 years.

### CMR acquisition for healthy human subjects

At all three participating institutions, CMR was performed using a 3-Tesla (T) system (Siemens 3T Prisma^fit^ for Institution A, Siemens 3T Verio for Institution B, and Siemens 3T SKYRA for Institution C). The CMR acquisition parameters for cine imaging and T1 mapping are described in Supplementary Material. To assess left ventricular (LV) myocardial function and mass, short-axis images of the LV were acquired using a cine balanced steady-state free precession (bSSFP) sequence [16]. Three short-axis Modified Look-Locker Inversion-recovery (MOLLI) images at the base, mid-cavity, and apex were acquired for native T1 mapping [16, 17]. Then, a total dose of 0.1 mmol/kg gadolinium agent (Uniray, gadoterate meglumine, Dongkook Pharmaceutical Co., Ltd.) was injected. Ten minutes after contrast injection, post-contrast MOLLI T1 mapping was acquired for T1 determination in an identical location as for native T1 mapping.

### Image acquisition for T1 mapping and ECV standardization phantom

**Fig. 2:**
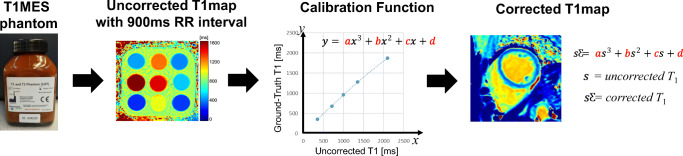
Diagram for the T1 map correction process. A T1 map was scanned with a T1MES phantom. The T1 value of the T1MES phantom provided by the manufacturer was considered as ground-truth T1, and the correction function was calculated based on the polynomial regression between the acquired T1 value and the ground-truth T1 value. The correction function was applied to the T1 maps of healthy human subjects to reduce the variation in T1 measurement. T1MES, T1 Mapping and ECV Standardization

We developed a novel method using the T1MES phantom. The process of image acquisition and T1 correction based on phantom are shown in Fig. 2. The T1MES phantom was scanned at each institution within a month in December 2019, when the clinical study started. The phantom was employed to measure the error in T1 measurement in three institutions with the MOLLI T1 mapping protocol based on the default MOLLI protocol provided by the CMR manufacturer (Siemens Healthineers) with adjustment of field of view and without other voluntary modification of scan parameters.

To measure the gold-standard T1 (T1_GS_) of the T1MES phantom in each institution, the inversion-recovery prepared turbo spin-echo [9] and the MOLLI sequence were used (Supplementary Table 1). Other scan considerations, such as setting the position in the iso-center, shim volume, and simulation ECG, were according to the instruction of the T1MES manual [18]. The ground-truth T1 (T1_GT_) of this T1MES phantom was regarded as the T1 described in the manual by the manufacturer.

### Phantom-based T1 correction

To reduce the variation in the T1, three correction methods were considered (Supplementary Table 2). First, a gold-standard T1-based correction function (GC) was calculated by multiple polynomial regression with T1_GS_ and T1_GT_ of the T1MES phantom. Second, a MOLLI T1-based correction function (MC) was calculated with the T1 on MOLLI (T1_ML_) and T1_GT_. Last, an internal reference T1-based correction function (IC) was calculated with the T1_ML_ and the T1_GS_. In particular, the MC and IC methods were subdivided according to whether the RR interval (RRI) of the subject was considered. When the RRI of each subject was considered (adaptive RRI), the correction function was calculated using a T1_ML_ with an RRI close to the RRI of the subject (Supplementary Table 3). Otherwise, a T1_ML_ with an RRI of 900 ms (static RRI) was used (Supplementary Table 4).

First-, second-, and third-order correction equations were applied to each correction method as follows:

$$ T1c=c\cdotp T1u+d $$

$$ T1c=b\cdotp T1{u}^2+c\cdotp T1u+d $$

$$ T1c=a\cdotp T1{u}^3+b\cdotp T1{u}^2+c\cdotp T1u+d $$

where T1u is the uncorrected T1, T1c is the corrected T1, and *a*, *b*, *c,* and *d* are coefficients of the correction function according to each correction method (Supplementary Table 3 and 4). We chose the equation that showed the lowest coefficient of variation (CoV, standard deviation/mean) after correction.

### CMR analysis

CMR images were anonymized and analyzed independently by two experienced observers (Y.J.S. and B.W.C., cardiac radiologists with 8 and 21 years of CMR experience, respectively) who were blinded to the clinical data. Cine imaging and T1 map images were analyzed using commercial software (cvi42 image analysis software, Circle Cardiovascular Imaging Inc.) (Supplementary Methods).

On cine bSSFP images, the endocardial and epicardial contours of the LV were semi-automatically drawn with manual adjustments when needed. LV end-diastolic volume (EDV) and end-systolic volume (ESV) were calculated using the modified Simpson method and were indexed to body surface area. LV ejection fraction was calculated as (EDV-ESV)/EDV.

Native and post-contrast T1 map images were generated by fitting pixels to the equation *s*(*t*) = *a* – *b* exp. (*t*/T1*), and T1 = T1*((*b*/*a*−1), where *a* and *b* are constants, *t* is time, and *s(t)* is the signal intensity at time *t*. On T1 map images, endocardial and epicardial contouring of the LV was performed semi-automatically, and manual adjustments were applied when needed. Native and post-contrast blood T1 times were measured on a region of interest drawn in the center of the blood pool. Native and post-contrast T1 values were measured in 16 AHA segments in the short-axis view of LV [19]. The myocardial ECV was calculated using the following equation [20]:

$$ \mathrm{ECV}=\left(1-\mathrm{hematocrit}\right)\cdotp {\left[\left(1/{\mathrm{T}1}_{\mathrm{myocardium}\ \mathrm{post}}\right)-\left(1/{\mathrm{T}1}_{\mathrm{myocardium}\ \mathrm{pre}}\right)\right]}_{/}\ \left[\left(1/{\mathrm{T}1}_{\mathrm{blood}\ \mathrm{post}}-1/{\mathrm{T}1}_{\mathrm{blood}\ \mathrm{pre}}\right)\right] $$

Native T1 and ECV fraction were analyzed as the mean of the 16 entire segments (global) and mid septum, after the exclusion of segments with image artifacts (e.g., off-resonance or partial volume artifact) that caused significant deterioration in T1 measurement.

### Statistical analysis

Statistical analyses were performed using MedCalc for Windows version 19.1.0.0 (MedCalc Software) and R (version 4.0.2, R Foundation). Continuous variables are expressed as the mean ± standard deviation or median with 25^th^ to 75^th^ percentile, and categorical variables are shown as counts and percentages. Categorical variables were compared using the *χ*^2^ or Fischer’s exact test. Continuous variables were compared among groups using ANOVA for normally distributed data and the Kruskal-Wallis test for non-normally distributed data. T1 mapping results were excluded from the analysis if measured T1 values were estimated as outliers by the Tukey method [21, 22]. Variations in native T1 and ECV fraction among institutions were compared before and after phantom-based correction using a CoV. Inter-observer reproducibility of T1 times and ECV was assessed using an intraclass correlation coefficient. A probability value less than 0.05 was considered statistically significant, and a Bonferroni-adjusted post hoc probability value of less than 0.02 was considered statistically significant for comparison between the three institutions.

## Results

### Study population

The baseline characteristics of the 71 subjects are shown in Table 1. No subject had a history of stroke, chronic obstructive pulmonary disease, or sleep apnea syndrome or presented hemochromatosis or anemia on the blood test.

**Table 1:** Patient characteristics

	Entire population (*n* = 71)	Institution A (*n* = 29)	Institution B (*n* = 16)	Institution C (*n* = 26)	*p* value
Sex (M:F)	34: 37	13:16	7:9	14:12	0.745
Mean age (years)	45.5 ± 15.5	48.2 ± 15.1	40.8 ± 15.6	45.5 ± 15.8	0.5
Age groups (M:F)					
20–29 years	14 (7:7)	4 (2:2)	4 (2:2)	6 (3:3)	
30-–39 years	14 (7:7)	4 (2:2)	6 (3:3)	4 (2:2)	
40–49 years	14 (7:7)	8 (3:5)	2 (2:0)	4 (2:2)	
50–59 years	14 (7:7)	6 (4:2)	2 (0:2)	6 (3:3)	
60–79 year	15 (6:9)	7 (2:5)	2 (0:2)	6 (4:2)	
Height (cm)	166.5 ± 7.9	165.2 ± 8.4	166.7 ± 7.3	167.7 ± 7.9	0.247
Weight (kg)	63.7 ± 10.6	62.8 ± 10.3	66.7 ± 10.8	63.0 ± 10.9	0.927
Body mass index (kg/m^2^)	22.9 ± 2.6	23.0 ± 2.2	24.0 ± 3.3	22.2 ± 2.4	0.292
Smoking history	14 (19.7)	8 (27.6)	3 (18.8)	3 (11.5)	0.326
Family history of cardiovascular disease	10 (12.3)	9 (31.0)	1 (6.2)	0 (0)	0.003
Systolic blood pressure (mmHg)	118.6 ± 13	122.7 ± 8.9	106.1 ± 11.6	121.8 ± 13.7	0.737
Diastolic blood pressure (mmHg)	75.7 ± 8	80.1 ± 7.2	70.2 ± 8.1	74.2 ± 7.9	0.007
Total cholesterol (mg/dL)	184.5 ± 31	189.9 ± 34.6	168.9 ± 24.3	187.9 ± 28.2	0.768
Creatinine (mg/dL)	0.8 ± 0.2	0.8 [0.7; 0.9]	0.8 [0.6; 0.8]	0.8 [0.7; 0.9]	0.625
NT_proBNP (pg/mL)	39.2 ± 40.6	46.8 ± 41.0	43.7 ± 45.5	28.1 ± 35.8	0.01
Hematocrit (%)	42.3 ± 3.7	41.4 ± 4.1	43.1 ± 3.5	42.7 ± 3.1	0.172

*M*, male; *F*, female; *eGFR*, estimated glomerular filtration rate; *NT_proBNP*, N-terminal pro-brain natriuretic peptide; *ECG*, electrocardiogram

The CMR examination was successfully completed in all subjects, but 2 patients (a 54-year-old male in institution A and a 29-year-old female in institution B) were excluded from the analysis of T1 mapping because their measured T1 values were estimated as outliers due to image artifacts. All participants had normal LV function on cine imaging (Supplementary Table 5). Artifacts precluded analysis of native T1 in 67 segments of 25 subjects and 83 segments of 35 subjects in post-contrast T1 maps, resulting in missing ECV in 111 segments of 41 subjects. Inter-observer agreement of measurements on T1 map was excellent for both native T1 and ECV (intraclass correlation coefficient 0.993 [95% confidence interval 0.989–0.996] and 0.998 [95% confidence interval 0.997–0.999]), respectively).

### Normal T1 values before and after phantom-based correction

**Fig. 3:**
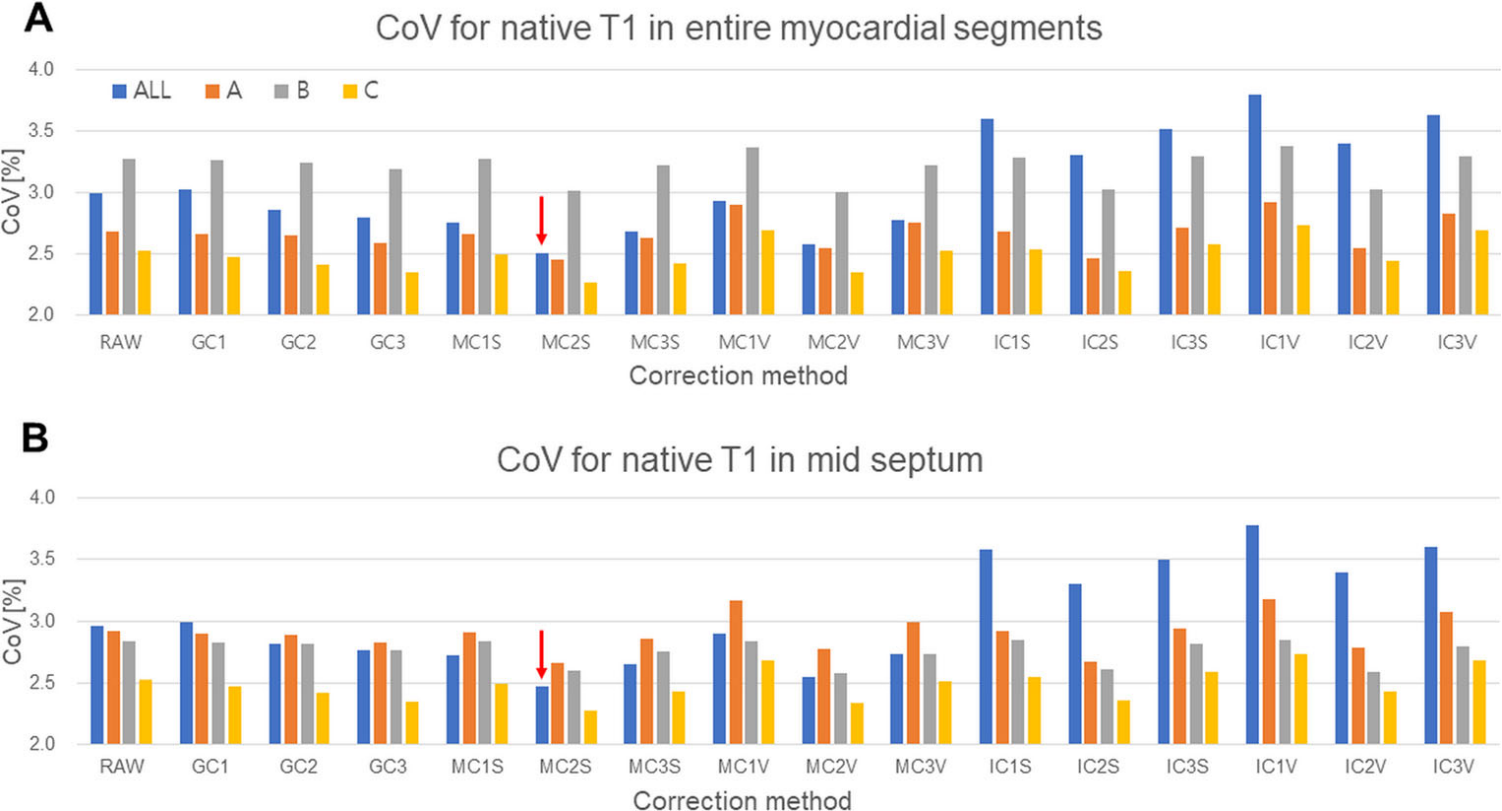
CoV of the native T1 map using various correction methods. The MC_2S_ (MOLLI T1 map-based second-degree correction function with static 900 ms RR interval) method had the lowest CoV compared to other correction methods on the (**A**) entire myocardial segments and (**B**) mid septum. The CoV decreased from 3.0 to 2.5% on the entire myocardial segments and from 2.96 to 2.48% on the mid septum (red arrow). CoV, coefficient of variation; MOLLI, Modified Look-Locker Inversion-recovery

The MC_2_ method (second-order correction equation with MC method) with static RRI was used for the correction of native and post-contrast T1 map images because the method showed the greatest decrease in the CoV of native T1 map in healthy human subjects among various correction methods (Fig. 3). Correction equations for myocardial native T1 and post-contrast T1 map for each institution are provided in Table 2.

**Table 2:** Correction equations using the MOLLI T1 map-based correction method (MC)

T1 map	Institution	Correction equation*	Coefficient of determination (*R*^2^)
For native T1 map, Second-degree MC with 900 ms RRI	A	*T*1*c* = − 0.0001376*T*1*u*^2^ + 1.307912*T*1*u* − 85.43315	0.9982
	B	*T*1*c* = − 0.0001273*T*1*u*^2^ + 1.288508*T*1*u* − 83.56056	0.9984
	C	*T*1*c* = − 0.0001360*T*1*u*^2^ + 1.278690*T*1*u* − 78.19730	0.9984
For post-contrast T1 map, Second-degree MC with 900 ms RRI	A	*T*1*c* = − 0.0000965*T*1*u*^2^ + 1.354858*T*1*u* − 109.29932	0.9964
	B	*T*1*c* = − 0.000072*xT*1*u*^2^ + 1.309827*T*1*u* − 97.524167	0.9967
	C	*T*1*c* = − 0.000103*T*1*u*^2^ + 1.337582*T*1*u* − 104.943758	0.9969

*The *T1u* is an input source of uncorrected T1 value, and *T1c* is the corrected T1 value

*MOLLI*, Modified Look-Locker Inversion-recovery; *RRI*, RR interval

The mean global native T1 and ECV fraction in 69 subjects are shown in Table 3. Mean RRI during the acquisition of native T1 and post T1 mapping sequences was 918.8 ± 144.1 ms (range 659.0–1261.3 ms) and 922.3 ± 129.7 ms (range 714.3–1247.5 ms), respectively. Before the phantom-based correction, the global native T1 was significantly different between the three institutions (1198.7 ± 32.1 ms for institution A, 1217.7 ± 39.9 ms for institution B, and 1232.7 ± 31.1 ms for institution C; *p* = 0.002), but ECV was not significantly different between the institutions (26.6 ± 1.8% for institution A, 27.5 ± 3.6% for institution B, and 27.4 ± 2.5% for institution C; *p* = 0.355). After phantom-based correction, the global native T1 was not significantly different between the three institutions (1284.5 ± 31.5 ms for institution A, 1296.5 ± 39.1 ms for institution B, and 1291.3 ± 29.3 ms for institution C; *p* = 0.440), and ECV was also not significantly different between the institutions (24.4 ± 2.2% for institution A, 25.9 ± 3.7% for institution B, and 25.4 ± 2.6% for institution C; *p* = 0.078). The mean of the corrected native T1 and ECV in 69 subjects were 1289.4 ± 32.4 ms and 25.0 ± 2.7%, respectively. After phantom-based correction, the CoV for the native T1 between the three institutions decreased from 3.0 to 2.5%.

**Table 3:** Comparison of normal native T1 values and ECV fractions between the three institutions

	Before phantom-based correction								After phantom-based correction							
	Entire population (*n* = 69)	Institution A (*n* = 28)	Institution B (*n* = 15)	Institution C (*n* = 26)	*p* value (between 3 groups)	*p* value (between A and B)	*p* value (between A and C)	*p* value (between B and C)	Entire population (*n* = 69)	Institution A (*n* =28)	Institution B (*n* = 15)	Institution C (*n* = 26)	*p* value (between 3 groups)	*p* value (between A and B)	*p* value (between A and C)	*p* value (between B and C)
RRI, native T1 (ms)	918.8 ± 144.1	943.4 ± 141.6	928.6 ± 133.1	885.3 ± 152.2	0.139	0.734	0.148	0.354	**-**	**-**	**-**	**-**	**-**			
RRI, post T1 (ms)	922.3 ± 129.7	936.6 ± 129.0	948.3 ± 119.1	890.3 ± 134.9	0.196	0.767	0.199	0.165	**-**	**-**	**-**	**-**	**-**			
Native T1 (ms)																
Entire myocardium	1215.7 ± 36.4	1198.7 ± 32.1	1217.7 ± 39.9	1232.7 ± 31.1	**0.002**	0.097	**< 0.001**	0.189	1289.4 ± 32.4	1284.5 ± 31.5	1296.5 ± 39.1	1291.3 ± 29.3	0.440	0.284	0.421	0.632
Mid septum	1226.9 ± 36.4	1210.7 ± 35.0	1227.1 ± 34.8	1244.2 ± 31.5	**0.004**	0.151	**< 0.001**	0.113	1300.5 ± 32.2	1296.2 ± 34.2	1305.6 ± 33.9	1302.1 ± 29.7	0.498	0.394	0.502	0.733
ECV (%)																
Entire myocardium	27.1 ± 2.5	26.6 ± 1.8	27.5 ± 3.6	27.4 ± 2.5	0.210	0.344	0.149	0.917	25.0 ± 2.7	24.4 ± 2.2	25.9 ± 3.7	25.4 ± 2.6	0.084	0.105	0.045	0.616
Mid septum	27.3 ± 2.4	26.7 ± 1.9	27.4 ± 3.1	27.8 ± 2.5	0.079	0.430	0.058	0.604	25.1 ± 2.6	24.2 ± 1.9	25.6 ± 3.1	25.8 ± 2.7	0.056	0.132	0.016	0.854

Boldface type indicates that the difference was statistically significant.

*ECV*, extracellular volume fraction; *RRI*, RR interval

In the measurement of the mid septum, native T1 was significantly different between the three institutions (1210.7 ± 35.0 ms for institution A, 1227.1 ± 34.8 ms for institution B, and 1244.2 ± 31.5 ms for institution C; *p* = 0.004) before phantom-based correction, but ECV was not significantly different between the institutions (26.7 ± 1.9% for institution A, 27.4 ± 3.1% for institution B, and 27.8 ± 2.5% for institution C; *p* = 0.079). After phantom-based correction, the mean native T1 of the mid septum was not significantly different between the three institutions (1296.2 ± 34.2 ms for institution A, 1305.6 ± 33.9 ms for institution B, and 1302.1 ± 29.7 ms for institution C; *p* = 0.498), and ECV was also not significantly different between the institutions (24.2 ± 1.9% for institution A, 25.6 ± 3.1% for institution B, and 25.8 ± 2.7% for institution C; *p* = 0.056). After phantom-based correction, the CoV for the native T1 between the three institutions decreased from 3.0 to 2.6%.

## Discussion

Our study demonstrates that the phantom-based T1 correction method can reduce the inter-institutional variation in native T1 and ECV measurements on myocardial T1 mapping. After phantom-based correction, the global native T1 on 3-T CMR is 1289.4 ± 32.4 ms, and the ECV was 25.0 ± 2.7% for healthy Korean subjects.

Previous studies have reported normal reference ranges of native T1 and ECV in healthy subjects on CMR obtained at individual institutions using 1.5-T [23–27] or 3-T scanners [14, 27, 28]. Due to the variety of factors affecting the T1 measurements, the published normal reference ranges of native T1 are heterogeneous among studies [6, 7]. Therefore, it is difficult to benchmark those values across different institutions. Moreover, the variations in measurements have hindered multi-institutional studies for myocardial T1 mapping, and such multi-institutional studies can be conducted only when all the institutions in the study have a uniform imaging setup with the same type of scanner and pulse sequence [2, 27].

Compared to previous studies regarding the reference T1 on CMR, our study has two main strengths. First, we reported the myocardial T1 in healthy Korean subjects with a sufficient sample size (*n* = 71). As ethnicity may affect the myocardial T1 and ECV similarly as it affects the LV volume and mass [29], defining normal reference ranges in the Korean population will help develop future studies to investigate CMR T1 mapping. Although some previous studies have reported native T1 and ECV in healthy volunteers in Korea [30–33], the studies conducted thus far comprised relatively small control groups (*n* < 30) for comparison against patients with cardiovascular disease. Second, this is a multi-institutional study conducted in three different institutions with different scanner types and provides standardized normal native T1 and ECV by using the phantom-based correction method. After phantom-based correction was applied, the differences in the mean native T1 among institutions became insignificant, and the CoV also decreased. Our results suggest that the phantom-based correction method is effective for the standardization of myocardial T1 achieved by reducing measurement variation.

We suggest that the phantom-based T1 correction method can reduce the variation in T1 measurement. Since the T1MES phantom program was established [9], repeatability of T1 measurement using the T1MES phantom has been demonstrated across centers with different field strengths, sequences, and scanners [12], and the T1MES phantom has been found to be useful for quality assurance in a multi-center setting. We employed this well-established phantom for the correction of the myocardial T1. For this purpose, we considered three types of correction methods (systemic, reference, and internal) with combinations of heart rate condition (static or adaptive RRI) and degree of correction equations (first-, second-, and third-degree equations). After several preliminary examinations, we concluded that the phantom-based T1 correction is feasible, and the MC method with a second-degree equation and static RRI was the most suitable for T1 correction because this method reduced variation among measurements made in the three participating institutions to the greatest extent. We expect that values obtained from different institutions might be interchangeable if our method would be validated in other sites with CMR scanners from different vendors.

Our study has several limitations. First, we included subjects from three institutions with different types of CMR scanners, but all of them were 3-T scanners from the same vendor and used the same MOLLI sequence. It is necessary to validate our findings using scanners of other vendors, pulse sequences, and 1.5-T to expand the application of our method. Second, it is unclear whether T1 variations among institutions originate from inter-subject variations or other factors, such as the CMR scanner, because different individuals were included from each institution. To validate our methods and exclude the effect of inter-subject variation, acquiring T1 in the same subject with scanners from multiple vendors and based on pulse sequences are needed. Finally, the time interval of blood sampling for hematocrit measurement between CMR acquisition was longer than 1 day in 57.7% (41 of 71) subjects, which might have diminished the reliability of ECV calculation [1]. However, some studies suggested that hematocrit measured on a different day from CMR could be useful for ECV calculation without a significant difference in the calculated value [34]. We assumed that our results for ECV may not have significantly deviated from those obtained if the hematocrit had been measured on the same day as CMR.

In conclusion, the standardized reference value of myocardial native T1 and ECV in healthy Korean subjects can be provided using the phantom-based correction method as it reduces variation in T1 measurement. Our phantom-based correction method may allow standardization of myocardial T1, which can facilitate the application of T1 mapping in multi-institutional, large-scale clinical trials.

## Supplementary Information

ESM 1 (DOCX 56 kb)

## Abbreviations

CMR: Cardiac magnetic resonance imaging
CoV: Coefficient of variation
ECG: Electrocardiogram
ECV: Extracellular volume
eGFR: Estimated glomerular filtration rate
LV: Left ventricular
MOLLI: Modified Look-Locker Inversion-recovery
RRI: RR interval
T1MES: T1 Mapping and ECV Standardization

## Declarations

### Guarantor

The scientific guarantor of this publication is BWC.

### Statistics and biometry

No complex statistical methods were necessary for this paper.

### Methodology

• prospective

• experimental

• multicenter study
