# Supplementary material for: Phantom-based correction for standardization of myocardial native T1 and extracellular volume fraction in healthy subjects at 3-Tesla cardiac magnetic resonance imaging
Source: Eur Radiol. 2022 Jun 30;32(12):8122–30. doi: 10.1007/s00330-022-08936-8 (PMC9705515; doi:10.1007/s00330-022-08936-8)
Supplement: Supplementary file 1 — (DOCX 56 kb) [file 330_2022_8936_MOESM1_ESM.docx]

**Supplementary Materials**

**Supplementary Methods**

**CMR acquisition protocols**

At all three participating institutions, CMR was performed using a 3-Tesla (T) system (Siemens 3T Prisma^fit^ for Institution A, Siemens 3T Verio for Institution B, and Siemens 3T SKYRA for Institution C). Short-axis images of the LV were acquired using a cine balanced steady state free precession (bSSFP) sequence, using a slice thickness of 8, 8, and 6 mm with a gap of 2, 0, and 4 mm in Institution A, Institution B, and Institution C, respectively. Three short-axis Modified Look-Locker Inversion-recovery (MOLLI) images at the base, mid-cavity, and apex were acquired for native T1 mapping, using an 8 image, 11 heart-beat 5-(3)-3 bSSFP sequence for all three institutions with slice thickness of 8 mm. Then, a total dose of 0.1 mmol/kg gadolinium agent (Uniray, gadoterate meglumine, Dongkook Pharmaceutical Co., Ltd.) was injected. Ten minutes after contrast injection, post-contrast MOLLI T1 mapping was performed for T1 determination at three slices in an identical location as for native T1 mapping, using a 9 image, 11 heart-beat 4-(1)-3-(1)-2 bSSFP sequence for all three institutions. Motion correction was applied to native T1 and post-contrast T1 mapping images.

**Supplementary Tables**

Table 1. T1 mapping parameters for phantom

| T1 mapping | Gold standard T1 map | Native T1 map | Post T1 map |
| --- | --- | --- | --- |
| Pulse sequence | Inversion Recovery prepared Turbo Spin Echo (IR-TSE) | MOLLI 5(3)3 | MOLLI 4(1)3(1)2 |
| Imaging sequence | Turbo Spin Echo | Trufisp | |
| TR/TE [ms] | 1000/9.3 | 306/1.1 | |
| FA [deg] | 180 | 35 | |
| Acceleration factor | Echo Train Length=2 | iPAT = Grappa, Acc=2 | |
| Matrix | 192 x 132 | 256 x 218 | |
| FOV [mm] | 300 x 206 | 360 x 306 | |
| Inversion time [ms] | 100, 200, 400, 800, 1200, 1600, 3000, 6000 | Initial TI = 100  Incremental TI = 80 | |
| RR interval [ms] | N/A | 700, 800, 900, 1000, 1100 | |

Table 2. Correction equations for T1

| Method | RRI [ms] | Input Source | Correction equation* | Index |
| --- | --- | --- | --- | --- |
| Gold standard T1 map-based correction (GC) | N/A | $T1u = {T1}_{GS}$  $T1c = {T1}_{GT}$ | $T1c=cT1u+d$ | ${GC}_{1}$ |
|  |  |  | $T1c=b{T1u}^{2}+cT1u+d$ | $GC_{2}$ |
|  |  |  | $T1c=a{T1u}^{3}+b{xT1u}^{2}+cx+d$ | ${GC}_{3}$ |
| MOLLI T1 map-based correction (MC) | 900 | $T1u= {T1}_{ML(S)}$  $T1c = {T1}_{GT}$ | $T1c=cT1u+d$ | ${MC}_{1S}$ |
|  |  |  | $T1c=b{T1u}^{2}+cT1u+d$ | ${MC}_{2S}$ |
|  |  |  | $T1c=a{T1u}^{3}+b{xT1u}^{2}+cx+d$ | ${MC}_{3S}$ |
|  | Various | $T1u = {T1}_{ML(V)}$  $T1c = {T1}_{GT}$ | $T1c=cT1u+d$ | ${MC}_{1V}$ |
|  |  |  | $T1c=b{T1u}^{2}+cT1u+d$ | ${MC}_{2V}$ |
|  |  |  | $T1c=a{T1u}^{3}+b{xT1u}^{2}+cx+d$ | ${MC}_{3V}$ |
| Internal reference-based correction (IC) | 900 | $T1u = {T1}_{ML(S)}$  $T1c = {T1}_{GS}$ | $T1c=cT1u+d$ | ${IC}_{1S}$ |
|  |  |  | $T1c=b{T1u}^{2}+cT1u+d$ | ${IC}_{2S}$ |
|  |  |  | $T1c=a{T1u}^{3}+b{xT1u}^{2}+cx+d$ | ${IC}_{3S}$ |
|  | Various | $T1u = {T1}_{ML(V)}$  $T1c = {T1}_{GS}$ | $T1c=cT1u+d$ | ${IC}_{1V}$ |
|  |  |  | $T1c=b{T1u}^{2}+cT1u+d$ | ${IC}_{2V}$ |
|  |  |  | $T1c=a{T1u}^{3}+b{xT1u}^{2}+cx+d$ | ${IC}_{3V}$ |

*The *x* is an input source of uncorrected T1 value, and *y* is the corrected T1 value.

Table 3. Correction coefficient with various RR intervals of myocardial native T1 map for the MOLLI T1 map-based correction method (MC)

| Institution | Method index | RRI [ms] | Coefficient of correction function | | | |
| --- | --- | --- | --- | --- | --- | --- |
|  |  |  | a | b | c | d |
| A | MC_1_ | 700 | 0 | 0 | 1.051E+00 | -3.304E+00 |
|  |  | 800 | 0 | 0 | 1.043E+00 | 1.156E+00 |
|  |  | 900 | 0 | 0 | 1.035E+00 | 5.357E+00 |
|  |  | 1000 | 0 | 0 | 1.029E+00 | 7.576E+00 |
|  |  | 1100 | 0 | 0 | 1.026E+00 | 9.989E+00 |
|  | MC_2_ | 700 | 0 | -1.252E-04 | 1.296E+00 | -8.470E+01 |
|  |  | 800 | 0 | -1.313E-04 | 1.302E+00 | -8.486E+01 |
|  |  | 900 | 0 | -1.376E-04 | 1.308E+00 | -8.543E+01 |
|  |  | 1000 | 0 | -1.418E-04 | 1.312E+00 | -8.678E+01 |
|  |  | 1100 | 0 | -1.481E-04 | 1.322E+00 | -8.903E+01 |
|  | MC_3_ | 700 | -1.995E-07 | 5.045E-04 | 7.314E-01 | 4.388E+01 |
|  |  | 800 | -1.881E-07 | 4.656E-04 | 7.639E-01 | 3.777E+01 |
|  |  | 900 | -1.804E-07 | 4.381E-04 | 7.870E-01 | 3.356E+01 |
|  |  | 1000 | -1.790E-07 | 4.324E-04 | 7.900E-01 | 3.280E+01 |
|  |  | 1100 | -1.821E-07 | 4.380E-04 | 7.875E-01 | 3.350E+01 |
| B | MC_1_ | 700 | 0 | 0 | 1.054E+00 | -9.291E+00 |
|  |  | 800 | 0 | 0 | 1.046E+00 | -4.517E+00 |
|  |  | 900 | 0 | 0 | 1.036E+00 | 9.215E-01 |
|  |  | 1000 | 0 | 0 | 1.032E+00 | 4.097E+00 |
|  |  | 1100 | 0 | 0 | 1.031E+00 | 5.195E+00 |
|  | MC_2_ | 700 | 0 | -1.057E-04 | 1.261E+00 | -7.798E+01 |
|  |  | 800 | 0 | -1.111E-04 | 1.264E+00 | -7.724E+01 |
|  |  | 900 | 0 | -1.274E-04 | 1.289E+00 | -8.356E+01 |
|  |  | 1000 | 0 | -1.314E-04 | 1.293E+00 | -8.317E+01 |
|  |  | 1100 | 0 | -1.378E-04 | 1.305E+00 | -8.657E+01 |
|  | MC_3_ | 700 | -1.895E-07 | 4.900E-04 | 7.278E-01 | 4.350E+01 |
|  |  | 800 | -1.895E-07 | 4.878E-04 | 7.260E-01 | 4.552E+01 |
|  |  | 900 | -1.765E-07 | 4.355E-04 | 7.788E-01 | 3.333E+01 |
|  |  | 1000 | -1.770E-07 | 4.342E-04 | 7.808E-01 | 3.422E+01 |
|  |  | 1100 | -1.843E-07 | 4.525E-04 | 7.697E-01 | 3.622E+01 |
| C | MC_1_ | 700 | 0 | 0 | 1.018E+00 | 7.368E+00 |
|  |  | 800 | 0 | 0 | 1.010E+00 | 1.242E+01 |
|  |  | 900 | 0 | 0 | 1.002E+00 | 1.522E+01 |
|  |  | 1000 | 0 | 0 | 9.961E-01 | 1.939E+01 |
|  |  | 1100 | 0 | 0 | 9.937E-01 | 1.949E+01 |
|  | MC_2_ | 700 | 0 | -1.277E-04 | 1.274E+00 | -7.902E+01 |
|  |  | 800 | 0 | -1.318E-04 | 1.276E+00 | -7.714E+01 |
|  |  | 900 | 0 | -1.360E-04 | 1.279E+00 | -7.820E+01 |
|  |  | 1000 | 0 | -1.447E-04 | 1.291E+00 | -8.076E+01 |
|  |  | 1100 | 0 | -1.488E-04 | 1.298E+00 | -8.424E+01 |
|  | MC_3_ | 700 | -1.684E-07 | 4.165E-04 | 7.760E-01 | 3.601E+01 |
|  |  | 800 | -1.551E-07 | 3.721E-04 | 8.121E-01 | 2.994E+01 |
|  |  | 900 | -1.530E-07 | 3.638E-04 | 8.172E-01 | 2.870E+01 |
|  |  | 1000 | -1.469E-07 | 3.374E-04 | 8.444E-01 | 2.294E+01 |
|  |  | 1100 | -1.401E-07 | 3.126E-04 | 8.691E-01 | 1.579E+01 |

Table 4. Correction coefficient of myocardial native T1 and post-contrast T1 map with 900 ms RR interval

| Correction method | T1 map | Method index | Institution | Coefficient of correction function | | | |
| --- | --- | --- | --- | --- | --- | --- | --- |
|  |  |  |  | a | b | c | d |
| Gold standard T1 map-based  correction  (GC) | IR-TSE | GC_1_ | A | 0 | 0 | 9.770E-01 | 7.528E+00 |
|  |  |  | B | 0 | 0 | 9.808E-01 | 5.505E+00 |
|  |  |  | C | 0 | 0 | 9.206E-01 | 2.295E+01 |
|  |  | GC_2_ | A | 0 | -6.234E-06 | 9.897E-01 | 3.284E+00 |
|  |  |  | B | 0 | -7.538E-06 | 9.961E-01 | 3.755E-01 |
|  |  |  | C | 0 | -3.446E-05 | 9.944E-01 | -2.735E+00 |
|  |  | GC_3_ | A | 5.520E-08 | -1.834E-04 | 1.151E+00 | -3.409E+01 |
|  |  |  | B | 4.178E-08 | -1.415E-04 | 1.118E+00 | -2.785E+01 |
|  |  |  | C | 5.046E-08 | -2.054E-04 | 1.158E+00 | -4.214E+01 |
| MOLLI T1 map-based correction  (MC) | Native T1 | MC_1_ | A | 0 | 0 | 1.035E+00 | 5.357E+00 |
|  |  |  | B | 0 | 0 | 1.036E+00 | 9.215E-01 |
|  |  |  | C | 0 | 0 | 1.002E+00 | 1.522E+01 |
|  |  | MC_2_ | A | 0 | -1.376E-04 | 1.308E+00 | -8.543E+01 |
|  |  |  | B | 0 | -1.274E-04 | 1.289E+00 | -8.356E+01 |
|  |  |  | C | 0 | -1.360E-04 | 1.279E+00 | -7.820E+01 |
|  |  | MC_3_ | A | -1.804E-07 | 4.381E-04 | 7.870E-01 | 3.356E+01 |
|  |  |  | B | -1.765E-07 | 4.355E-04 | 7.788E-01 | 3.333E+01 |
|  |  |  | C | -1.530E-07 | 3.638E-04 | 8.172E-01 | 2.870E+01 |
|  | Post T1 | MC_1_ | A | 0 | 0 | 1.180E+00 | -5.357E+01 |
|  |  |  | B | 0 | 0 | 1.179E+00 | -5.593E+01 |
|  |  |  | C | 0 | 0 | 1.146E+00 | -4.305E+01 |
|  |  | MC_2_ | A | 0 | -9.653E-05 | 1.355E+00 | -1.093E+02 |
|  |  |  | B | 0 | -7.223E-05 | 1.310E+00 | -9.752E+01 |
|  |  |  | C | 0 | -1.034E-04 | 1.338E+00 | -1.049E+02 |
|  |  | MC_3_ | A | -4.142E-07 | 1.108E-03 | 3.479E-01 | 1.104E+02 |
|  |  |  | B | -4.002E-07 | 1.089E-03 | 3.407E-01 | 1.137E+02 |
|  |  |  | C | -3.633E-07 | 9.763E-04 | 4.180E-01 | 9.839E+01 |
| Internal reference-based correction  (IC) | Native T1 | IC_1_ | A | 0 | 0 | 1.059E+00 | -1.736E+00 |
|  |  |  | B | 0 | 0 | 1.056E+00 | -4.362E+00 |
|  |  |  | C | 0 | 0 | 1.089E+00 | -8.298E+00 |
|  |  | IC_2_ | A | 0 | -1.428E-04 | 1.342E+00 | -9.595E+01 |
|  |  |  | B | 0 | -1.288E-04 | 1.311E+00 | -8.982E+01 |
|  |  |  | C | 0 | -1.173E-04 | 1.327E+00 | -8.886E+01 |
|  |  | IC_3_ | A | -2.507E-07 | 6.571E-04 | 6.182E-01 | 6.938E+01 |
|  |  |  | B | -2.309E-07 | 6.078E-04 | 6.443E-01 | 6.317E+01 |
|  |  |  | C | -2.432E-07 | 6.768E-04 | 5.938E-01 | 8.096E+01 |
|  | Post T1 | IC_1_ | A | 0 | 0 | 1.207E+00 | -6.174E+01 |
|  |  |  | B | 0 | 0 | 1.201E+00 | -6.205E+01 |
|  |  |  | C | 0 | 0 | 1.244E+00 | -7.093E+01 |
|  |  | IC_2_ | A | 0 | -1.012E-04 | 1.390E+00 | -1.202E+02 |
|  |  |  | B | 0 | -7.230E-05 | 1.332E+00 | -1.037E+02 |
|  |  |  | C | 0 | -7.316E-05 | 1.379E+00 | -1.147E+02 |
|  |  | IC_3_ | A | -5.079E-07 | 1.376E-03 | 1.551E-01 | 1.492E+02 |
|  |  |  | B | -4.709E-07 | 1.294E-03 | 1.921E-01 | 1.448E+02 |
|  |  |  | C | -4.907E-07 | 1.385E-03 | 1.372E-01 | 1.599E+02 |

Table 5. Comparison of ventricular functional parameters on CMR

|  | Entire population (n=71) | Institution A (n=29) | Institution B (n=16) | Institution C (n=26) | P value |
| --- | --- | --- | --- | --- | --- |
| LVEDV/BSA (mL/m^2^) | 69.4 ± 11.8 | 69.5 ± 12.5 | 69.8 ± 12.9 | 69.0 ± 10.8 | 0.881 |
| LVESV/BSA (mL/m^2^) | 27.0 ± 6.0 | 27.2 ± 6.6 | 27.9 ± 5.6 | 26.3 ± 5.7 | 0.593 |
| LVEF (%) | 61.1 ± 5.3 | 61.0 ± 5.3 | 59.9 ± 3.9 | 61.8 ± 6.0 | 0.61 |
| LV mass/BSA (g/m^2^) | 49.2 ± 11.0 | 49.1 ± 10.9 | 47.0 ± 11.9 | 50.6 ± 10.8 | 0.632 |
| RVEDV/BSA (mL/m^2^) | 76.1 ± 17.7 | 75.9 ± 16.6 | 76.3 ± 21.0 | 76.2 ± 17.6 | 0.962 |
| RVESV/BSA (mL/m^2^) | 32.9 ± 9.6 | 31.5 ± 9.1 | 35.9 ± 12.2 | 32.8 ± 8.3 | 0.604 |
| RVEF (%) | 56.7 ± 6.4 | 58.8 ± 5.4 | 52.8 ± 8.5 | 56.8 ± 5.2 | 0.235 |

CMR = cardiac magnetic resonance imaging; EDV = end-diastolic volume; BSA = body surface area; EF = ejection fraction; ESV = end-systolic volume; LV = left ventricle; RV = right ventricle
